# Adsorption of Cellular Proteins to Polyelectrolyte-Functionalized Gold Nanorods: A Mechanism for Nanoparticle Regulation of Cell Phenotype?

**DOI:** 10.1371/journal.pone.0086670

**Published:** 2014-02-06

**Authors:** Patrick N. Sisco, Christopher G. Wilson, Davin Chernak, Jessica C. Clark, Elissa M. Grzincic, Kayla Ako-Asare, Edie C. Goldsmith, Catherine J. Murphy

**Affiliations:** 1 Department of Chemistry, University of Illinois at Urbana-Champaign, Urbana, Illinois, United States of America; 2 Department of Cell Biology and Anatomy, University of South Carolina School of Medicine, Columbia, South Carolina, United States of America; University of California, Merced, United States of America

## Abstract

Cell behavior in the presence of nanomaterials is typically explored through simple viability assays, but there is mounting evidence that nanomaterials can have more subtle effects on a variety of cellular functions. Previously our lab demonstrated that gold nanorods functionalized with polyelectrolyte multi-layers inhibited rat cardiac fibroblast-mediated remodeling of type I collagen scaffolds by altering fibroblast phenotype and the mechanical properties of the collagen network. In this work, we examine a possible mechanism for these effects: adsorption of cellular proteins by the nanorods. Mass spectrometric and gel electrophoresis of media collected from cultured cells suggests that a number of proteins, some of which mediate cell-cell and cell-matrix interactions, adsorb onto the surface of these nanoparticles in vitro. Polyethylene glycol coating of the nanorods largely mitigates protein adsorption and fibroblast-mediated collagen remodeling. These results suggest that adsorption of proteins by nanorods could have a significant effect on cell functions, including fibroblast-mediated matrix remodeling.

## Introduction

Gold nanomaterials have received considerable attention for use in biomedical applications due to their unique optoelectronic properties [1]–[3] which make them ideal for research in cellular tracking [4], imaging [5], [6], biochemical sensing [1], drug delivery [7], [8], and therapeutics [9], [10]. In order to fruitfully study biomedical applications of gold nanomaterials, a fundamental understanding of how cells interact with and respond to an environment containing these nanomaterials is crucial. There have been multiple studies that document the cellular uptake and cytotoxicity of gold nanoparticles of various shapes in different cell types [11]–[16]. There have also been recent exciting studies that describe the emerging relationships between the surface chemistry of nanoparticles and serum protein adsorption (“the protein corona”) [17]–[26]; however, little is known about how protein adsorption to nanoparticles can affect cellular functions [24], [26]–[28] or interactions between the cells and their extracellular matrix (ECM). For many in vitro and in vivo applications, the local environment around the nanoparticles will be rich in soluble proteins (present in serum and interstitial fluid), insoluble ECM proteins such as collagen, and cell surface proteins.

Previously, we reported that gold nanorods coated with polyelectrolyte multilayers terminated with anionic poly(styrene sulfonate) (PSS) substantially altered the matrix-remodeling behavior of neonatal rat cardiac fibroblasts in type I collagen gels [28]. We observed that cardiac fibroblasts suspended in 3-dimensional collagen hydrogels doped with PSS-coated gold nanorods had lower expression of mRNAs encoding α-smooth muscle actin and collagen type I than controls that contained no nanomaterials; these mRNA changes correlate with the inability of fibroblasts to differentiate into a myofibroblasts phenotype. This important result indicated that biocompatible nanorods have the capacity to alter the phenotype of the cells in vitro [28]. This unexpected finding could be highly detrimental to biomedical applications of these materials; yet, if properly understood, may also provide a new way to intervene with cell fate and response in a beneficial way.

In addition to the effects nanorods have on cells themselves, possibly via the protein corona, nanorods also alter the physical properties of the matrix that surrounds them. We quantitatively examined the effects of polyelectrolyte-coated nanorods on the polymerization and mechanical properties of type I collagen hydrogels, frequently used as an *in vitro* model for tissue and also the native ECM of the cells in the phenotype study [29], [30]. Our results showed that anionic nanorods measurably altered the mechanical properties, network morphology, and polymerization kinetics of type I collagen, while cationic nanorods had only small effects on these properties. The physical mechanism by which these effects are manifested can once again be hypothesized to be due to the protein corona: proteins or other molecules that are adsorbed to nanomaterials change local concentrations, or conformations [31], and thus alter the nature of the ECM and how it formed.

Here, we report the composition of the protein corona adsorbed onto polyelectrolyte-coated gold nanorods from culture media conditioned by rat cardiac fibroblasts. We demonstrate differential binding of proteins to nanorods that bear positively or negatively charged functional groups. Furthermore, the hypothesis that adsorbed proteins actually cause behavior changes in fibroblasts was tested using “protein-resistant” coatings on the nanoparticles as functional controls. Reduced protein adsorption to these “resistant” particles was observed along with a reduction in nanomaterial-induced cellular behavior. Taken together, these experiments support the notion that the protein corona that surrounds nanomaterials provides a molecular mechanism to influence cell behavior.

## Methods

### Chemicals

Chloroauric acid (HAuCl_4_ **^.^**3H_2_0), sodium borohydride (NaBH_4_), ascorbid acid, poly(4-styrenesulfonic acid-*co*-maleic acid) sodium salt (PSS-MA; 1∶1 4-styrenesulfonic acid:maleic acid mole ratio, MW ∼20,000 g/mol), poly(sodium-4-styrenesulfonate; PSS, MW ∼70,000 g/mol), poly(diallyldimethylammonium chloride; PDADMAC, MW ∼15,000 g/mol), and ultrapure hexadecyltrimethylammonium bromide (CTAB) were obtained from Sigma-Aldrich (Sigma-Aldrich, St. Louis, MO). All other chemicals were purchased from Sigma unless otherwise noted and all solutions were prepared with 18 MΩ resistivity water using a Millipore Biocel ultrafiltration system (EMD Millipore; Billercia, MA).

### Instrumentation

Ultraviolet-visible spectroscopy was performed on a Cary 500 UV-Vis spectrophotometer (Agilent Technologies, Wilmington, DE). Transmission electron microscopy was performed on a JEOL 2100 Cryo-TEM instrument (JEOL, Peabody, MA). Light scattering and zeta potential analysis were performed on a Brookhaven Instruments ZetaPALS machine (Brookhaven Instrument Corp., Holtsville, NY).

### Gold Nanorod Synthesis and Polyelectrolyte Coating

Gold nanorods (408±97 nm long, 22±3 nm wide) were prepared in aqueous solution using a seed-mediated surfactant-directed approach previously described and purified by centrifugation and washing [32], [33]. Using layer-by-layer (LbL) assembly, (layers of oppositely charged polyelectrolytes PDADMAC and PSS) were deposited on the surface of the nanorods. The polymer coating was used to render the nanorods anionic (PSS) or cationic (PDADMAC) in aqueous solution, to mask the surfactant bilayer, and reduce potential toxicity. Polyethylene glycol (PEG) coated nanorods were prepared using PSS-MA in lieu of PSS and resuspended in 2-(N-morpholina)ethanesulfonic acid buffer (10 mM, pH 5.5) to which 0.1 M methoxy-PEG amine (5 k or 350 MW) was added. The reaction was allowed to proceed for 15 min after which 0.5 mg of 1-ethyl-3-[3-dimethylaminopropyl]carbodiimide hydrochloride (EDC; Pierce Chemical Company, Rockford, IL) was added and incubated overnight. Particles were purified by centrifugation as noted previously and resuspended in water.

### Protein Binding and Identification

Neonatal rat cardiac fibroblasts were isolated from 3–4 day old Sprague Dawley pups as previously described [52]. Animals were sacrificed by rapid decapitation under an animal use protocol approved by the University of South Carolina Institutional Animal Care and Use Committee. Neonatal cardiac fibroblasts were cultured in Dulbecco’s Modified Eagle Medium (DMEM) supplemented with 10% newborn bovine serum, 5% fetal bovine serum, 100 U/mL penicillin G, 100 µg/mL streptomycin, and 10 µg/mL gentamicin (Invitrogen, Carlsbad, CA). After 48 hours, media was collected for use in experiments (referred to as conditioned media). Nanorods (between 10^9^ and 10^10^ rods) were incubated in conditioned media overnight at 37°C followed by centrifugation to separate them from the media. After washing with phosphate buffered saline, bound proteins were eluted using 1 M NaCl, separated on a 4–20% gradient sodium dodecylsulfate–polyacrylamide gel electrophoresis (SDS-PAGE) gel (Thermo Fisher Scientific, Rockford, IL) and silver stained using the Silver Quest staining kit (Invitrogen) as per manufacturer’s instructions. To identify specific proteins bound to the nanorods, samples were sent to ProtTech (ProtTech, Norristown, PA) where they were reduced using dithiothreitol, alkylated with iodoacetamide (20 mM), trypsin digested, and analyzed by liquid chromatography-tandem mass spectrometry (LC-MS/MS) using a system with a high performance liquid chromatography reverse phase C18 column coupled to an ion trap mass spectrometer (LCQ Deca XP Plus; Thermo, Palo Alto, CA). Protein identification was determined using the Non-Redundant Protein Database from GeneBank with ProtTech’s ProtQuest software package.

### Collagen Gel Contraction Assays

Three dimensional collagen gels containing neonatal cardiac fibroblasts seeded with PSS, PDADMAC or PEGylated gold nanorods were prepared as previously described [28]. Briefly, pepsin-extracted acid-solubilized bovine type I collagen (Advanced BioMatrix, San Diego, CA) was neutralized by the addition of 0.2 N 4-(2-hydroxyethyl)-1-piperazineethanesulfonic acid (HEPES), pH 9.0 and 10X alpha minimal essential medium (αMEM) on ice at volumetric ratios of 8∶1: 1, respectively. For gels containing nanorods, 5.6×10^9^ nanorods/gel were re-suspended in the HEPES buffer prior to preparing the collagen mixture. Fibroblasts (200,000 cells/ml) were combined with the neutralized collagen and added in 100 µL aliquots to the wells of 96 well microtiter plates and allowed to polymerize for 1 hour at 37°C in a 5% CO_2_ humidified incubator. After polymerization, culture media was added to each well and the gels were manually detached from the wall of the well. Gels were photographed after 24 hours in culture with a Canon EOS Rebel digital camera and gel areas were measured using ImageJ. Statistical analysis was conducted using a Student’s T-test in Microsoft Excel.

### Analysis of Gene Expression Using Real Time PCR

Three gels per condition were pooled, solubilized in Trizol (Invitrogen) and RNA isolated using the RNeasy Micro RNA kit with on column DNase I digestion as recommended by the manufacturer (Qiagen, Valencia, CA). RNA concentrations were determined using a BioRad Experion system with standard sensitivity RNA chips and 50 ng of total RNA was used to prepare cDNA using the iScript cDNA synthesis kit (Bio-Rad Laboratories, Hercules, CA). PCR reactions were carried out using iQ Supermix (BioRad) with primers specific for rat **α**-smooth muscle actin (α-SMA) (forward: 5′ GGAGTGATGGTT-GGAATGG 3′; reverse: 5′ ATGATGCCGTGTTCTATCG 3′) and control gene acidic ribosomal phosphoprotein (ARBP) (forward: 5′ TAGAGGGT-GTCCGCAATG 3′; reverse: 5′ GAAGGTGTAGTCAGTC-TCC 3′). Data were analyzed using the fold change method incorporating primer efficiency with the REST2009 program (Qiagen Inc., Valencia, CA).

## Results and Discussion

### Role of Surface Charge in Mediating Nanorod-induced Changes in Cell Behavior

In our previous experiments, the presence of PSS-coated gold nanorods in fibroblast-seeded three-dimensional collagen gels led to reduced gel contraction by the fibroblasts and corresponding changes in gene expression [28], [29]. For these neonatal cardiac fibroblasts, the gene expression changes correspond to a switch between a myofibroblast phenotype and a fibroblast phenotype. If our hypothesis – that the adsorption of proteins to nanoparticles “shifts the cells’ equilibrium” causing them to maintain a fibroblast phenotype - has merit, then chemical functionalization of the particles to reduce protein adsorption should negate these observed effects. We used a number of assays, including collagen gel contraction assays, gene expression and protein binding, to compare the effects of nanorods whose surfaces terminated in negatively charged PSS or positively charged PDADMAC to nanorods whose surfaces terminated with PEG which is popularly considered to “resist” protein adsorption, depending on PEG conformation [34], [35].

**Figure 1:**
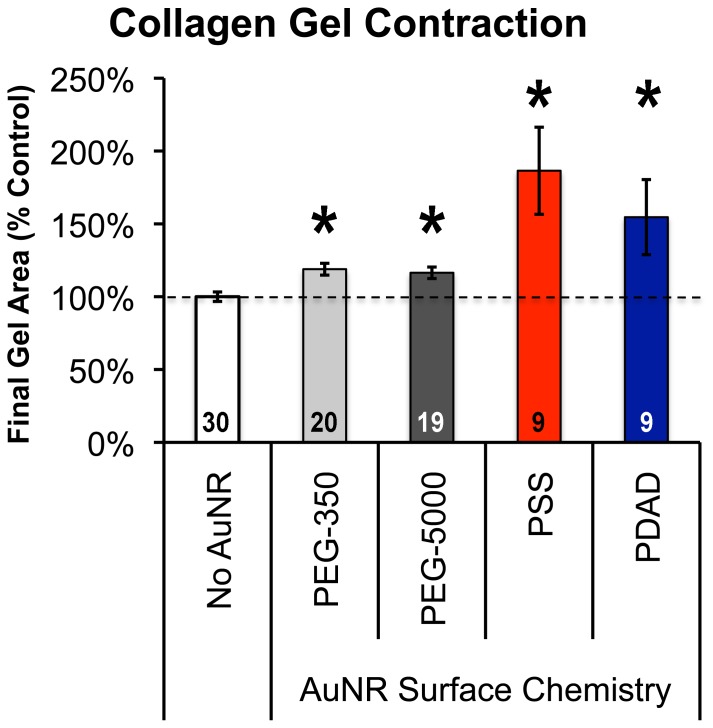
Surfaced-modified Gold Nanoparticles Alter Fibroblast Contractile Activity. Collagen gel areas were measured after 24 hours of culture for gels containing cardiac fibroblasts and gold nanorods with neutral (PEG), negatively charged (PSS) or positively charged (PDAD) surfaces. Larger bars indicate less contraction. Error bars represent standard error of the mean (sample sizes indicated within each bar) and statistically significant differences (p<0.05 as determined by Student’s T-test) compared to No AuNR controls are indicated by an asterisk.

**Figure 2:**
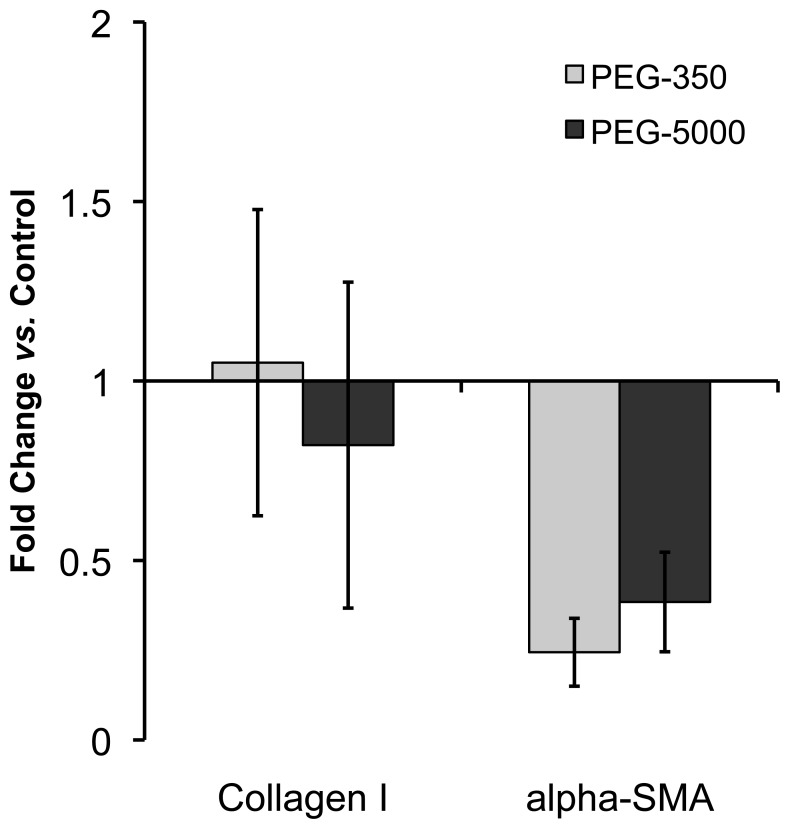
PEGylated Nanorods Have Minimal Effect on Fibroblast Gene Expression. The addition of PEGylated nanorods resulted in a small decrease in α-SMA expression. Fold change is the change in gene expression in PEG containing samples relative to no nanorod containing controls. Error bars are standard error of the mean (n = 3).

To confirm that protein adsorption was involved in mediating differences in collagen gel contraction, gels containing cardiac fibroblasts and 5.6×10^9^ PEGylated (350 or 5000 MW PEG) or PSS- or PDADMAC-coated rods/gel were prepared and allowed to contract for 24 hours [28]. As shown in Figure 1, collagen gels without nanorods exhibited the maximal level of contraction measured whereas the addition of nanorods, regardless of coating reduced the amount of fibroblast-mediated contraction. PEGylated nanorods triggered a modest, but significant, decrease in contraction compared to control gels consisting of fibroblasts without nanorods; however, this decrease was not as great as the inhibition in contraction observed with either PSS or PDADMAC coated nanorods after 24 hours. These data suggest that the presence of a protein reactive surface coating on the nanorods is critical for effecting profound changes in fibroblast-mediated collagen gel contraction. Polyelectrolyte-coated nanorod inhibition of collagen gel contraction has been associated with decreased differentiation of fibroblasts into a myofibroblast phenotype, evidenced at the gene level by a decrease in α-smooth muscle actin (α-SMA) expression [28]. To determine if the fibroblasts exposed to PEGylated nanorods differentiate into myofibroblasts, real time PCR was conducted. With the PEG-350 and PEG-5000 coated nanorods, α-SMA gene expression decreased very slightly (<0.4 fold in both cases) compared to control (no nanorod) gels (Figure 2), indicating that PEGylated nanorods did not significantly alter α-SMA gene expression in the cardiac fibroblasts and that these cells differentiated into myofibroblasts at similar levels compared to fibroblasts not exposed to nanorods. No change in type I collagen expression, a protein also produced at elevated levels by myofibroblasts, was observed following exposure to PEGlyated nanorods (Figure 2). In contrast, α-SMA gene expression decreased more than 5.1-fold in the presence of PSS-coated gold nanorods at the same concentration and a decrease in type I collagen mRNA was also detected [28].

### Characterization of Protein Adsorption onto Polyelectrolyte-Coated Gold Nanorods

**Figure 3:**
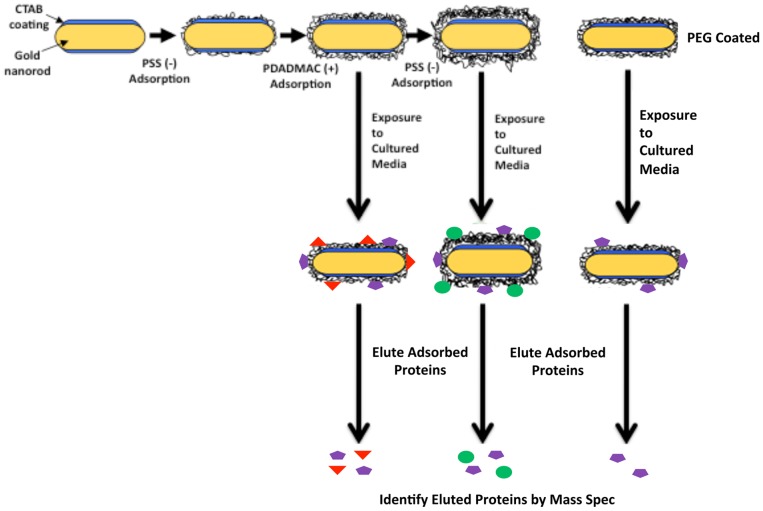
Schematic of Protein Isolation and Identification. Gold nanorods were prepared as described in the text and polymer-coated using layer-by-layer assembly, yielding nanorods coated with PDADMAC, PSS or PEG. After incubation with conditioned media, nanorods were centrifuged and proteins eluted with increasing salt concentrations. The resulting fractions were examined by SDS-PAGE and proteins visualized by silver staining, followed by mass spectrometry analysis.

**Figure 4:**
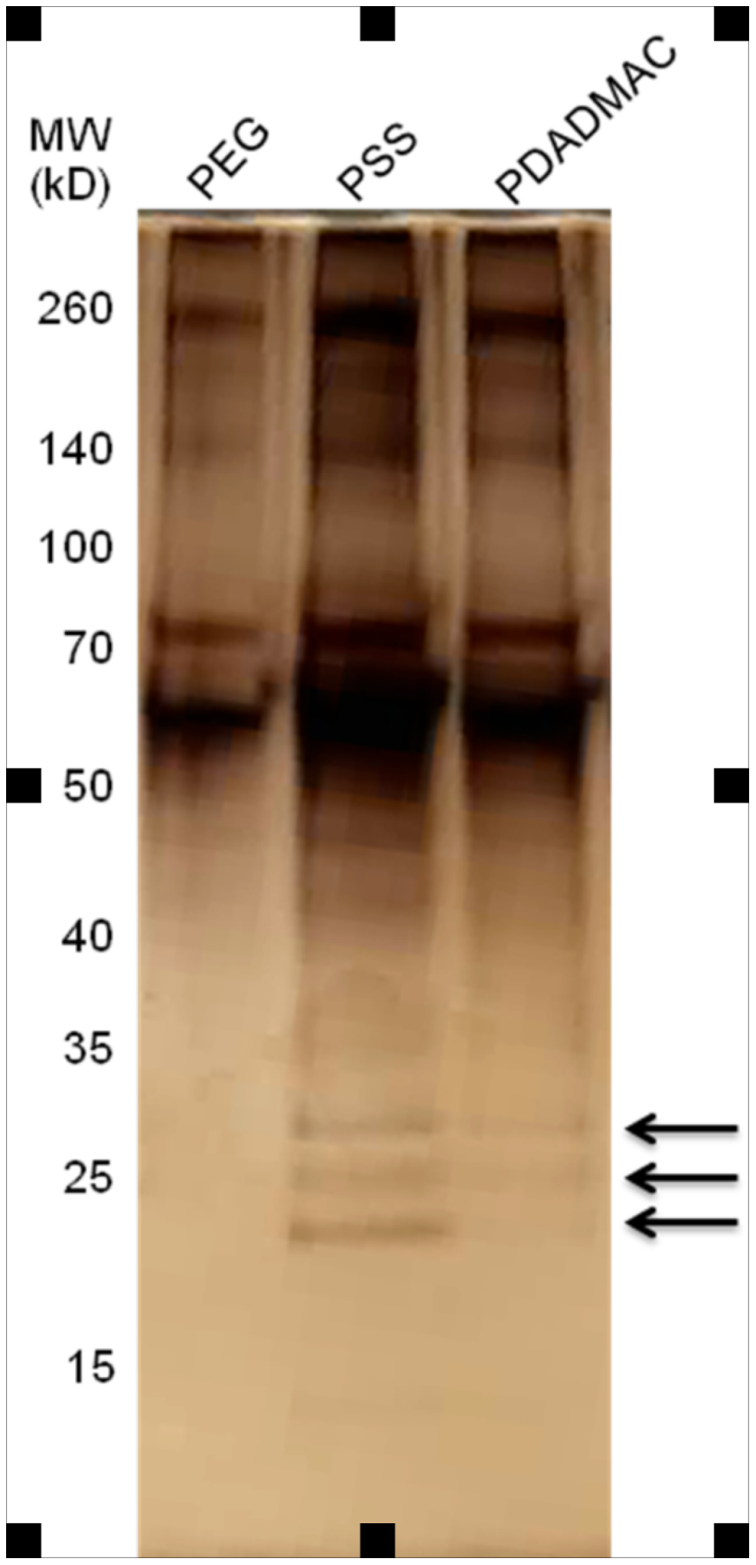
SDS-PAGE Comparison of Protein Adsorption. Protein binding profiles were compared between PEG-, PSS-, and PDADMAC-coated nanorods. Arrows indicate bands (20–30 kD range) in PSS/PDADMAC samples that were not observed in PEG samples.

To test our hypothesis that the protein binding characteristics of the nanoparticle coating are the mitigating factor in the observed changes in fibroblast behavior, PDADMAC-, PSS- and PEG-coated nanorods were exposed to fibroblast conditioned culture media and the adsorbed proteins identified. The procedure used to isolate and characterize nanorod-bound proteins is outlined in Figure 3. Briefly, polyelectrolyte-coated gold nanorods were exposed to fibroblast-conditioned culture media, bound proteins were eluted, and mass spectrometry was used to identify the proteins. Initial experiments employing varying salt concentrations (from 0.01–1 M NaCl) to elute bound proteins, analyzed by SDS-PAGE with silver staining, demonstrated no significant difference in banding pattern (data now shown), so all subsequent experiments to identify bound proteins were conducted using 1 M NaCl for protein elution. For these studies, conditioned media was obtained from neonatal rat cardiac fibroblasts, making it easy to differentiate proteins produced by the fibroblasts from bovine serum proteins present in the media. The incubation of nanorods with culture media resulted in significant protein adsorption as evident in Figure 4. Equal concentrations (10 µg/ml) of eluted protein samples were separated by SDS-PAGE and visualized by silver staining. Analysis of silver stained SDS-PAGE gels showed the presence of at least 5 protein bands ranging from ∼20 to 260 kDa in molecular weight, and 3 bands detected in PSS- and PDADMAC-terminated nanorods that were not evident in PEGylated nanorods.

**Figure 5:**
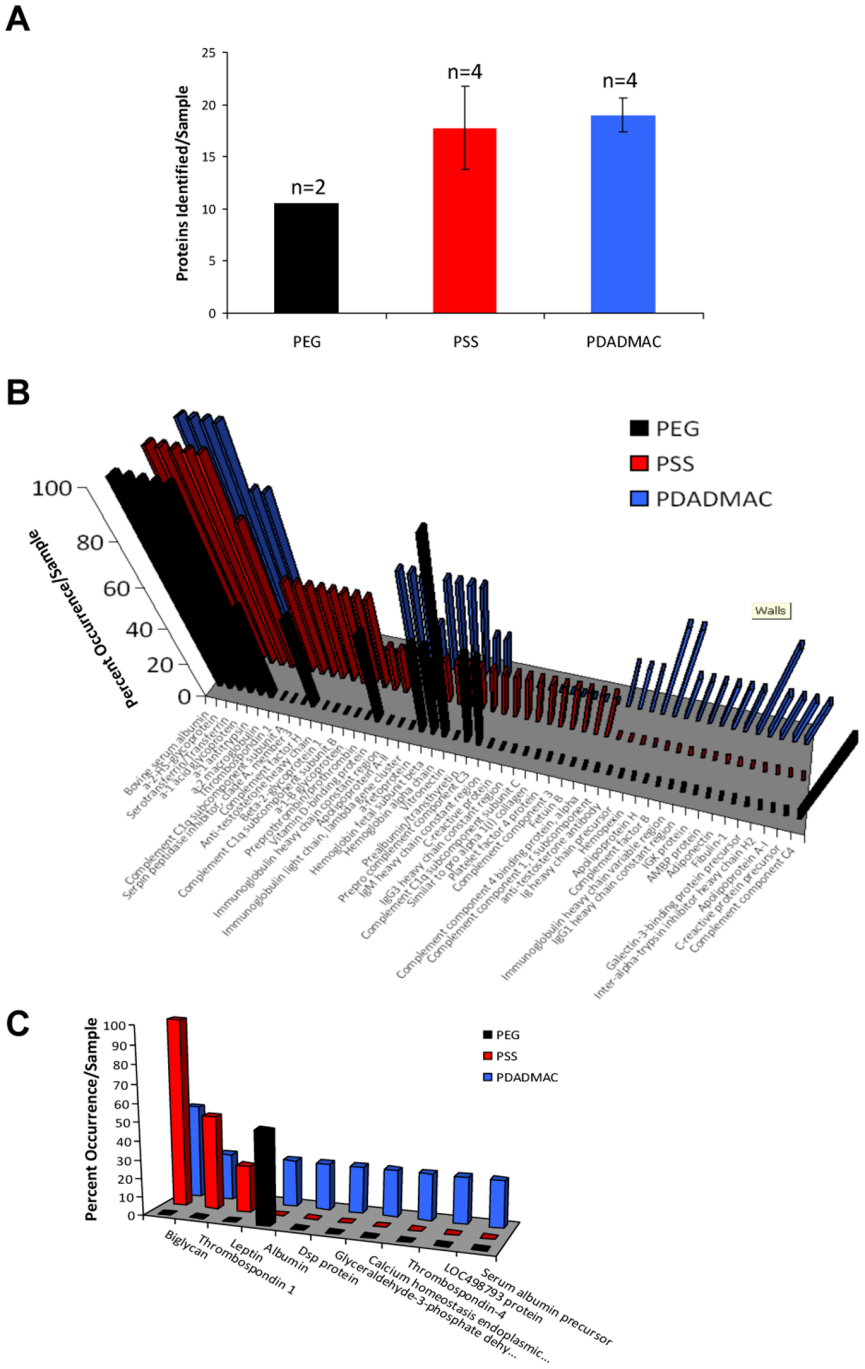
Identification of Proteins Extracted from PEG-, PSS-, and PDADMAC-coated Gold Nanorods. (A) Total number of proteins detected from each nanorods preparation. Data are mean +/− SEM. (B) Frequency of detection of unique bovine proteins, expressed as a percentage of the total number of samples analyzed, from each nanorods preparation. Data are mean +/− SEM. (C) Frequency of identification of unique rat proteins in each preparation of AuNR expressed as a percentage of the total number of samples analyzed. Data are means of n = 2–4 samples/group.

LC-MS/MS analysis of protein eluates revealed that the nanorods adsorbed a more diverse population of proteins than originally expected based on the electrophoresis data. On average, PSS- and PDADMAC-coated nanorods bound almost twice as many unique proteins as PEG-terminated nanorods (Figure 5A). Comparing Figures 5B and C, it is clear that the vast majority of proteins which adsorbed onto the nanorods were of bovine origin and that not all proteins detected adsorbed equally. The frequency of protein detection, determined by the number of times the protein was identified versus the total number of samples analyzed, demonstrates that some proteins preferentially adsorbed onto the nanorods. Bovine albumin, α-2-HS glycoprotein, serotransferrin/transferrin, and α-1 acid glycoprotein were by far the most frequently detected bovine proteins, found in all PSS-, PDADMAC-, and PEG-coated nanorod samples (Figure 5B). Whereas rat proteins accounted for only 6.7%, 8.1% and 17.8% of all proteins identified on PEG, PSS and PDADMAC coated nanorods respectively, with only one biomolecule, biglycan, beginning detected at 100% frequency (Figure 5C).

In a combination of four independent analyses, 37 unique proteins were identified which bound to the PSS-coated nanorods while 45 unique proteins adsorbed to PDADMAC-coated nanorods (Table S1). Of these proteins, 6 associated with the PSS nanorods and 4 with the PDADMAC nanorods were identified in all experiments (Table 1). In addition to those proteins identified in all experiments, α-2-macroglobulin (bovine origin) and thrombospondin 1 (once as bovine and twice as rat) were identified as binding to PSS-coated nanorods in 75% of the experiments; α-2-macroglobulin and α-1-antitrypsin, both of bovine origin, bound to PDADMAC-coated nanorods in 75% of the experiments. Interestingly, rat biglycan was also detected as binding to the PDADMAC-terminated nanorods in 50% of the experiments.

**Table 1:** Identification of Proteins Adsorbed onto Polyelectrolyte-coated Gold Nanorods.

PSS Coated Nanorods	PDADMAC Coated Nanorods
α-1 acid glycoprotein	α-1 acid glycoprotein
α-1 antitrypsin	α-2-HS glycoprotein
α-2-HS glycoprotein	Albumin
Albumin	Serotransferrin/Transferrin
*Biglycan*	
Serotransferrin/Transferrin	

The data in Tables S1 and 1 indicate that polyelectrolyte-coated gold nanorods can adsorb a wide variety of proteins; however, as indicated by the species of origin, most of these proteins were present in the serum added to the cell culture media, and not from the rat cardiac fibroblasts. In addition to the variety of proteins observed, an examination of the molecular weights of these proteins indicated that there was no significant difference in adsorption based on protein size (Figure S1). The average molecular weight of proteins adsorbed onto PSS- and PDADMAC-coated nanorods was 57 kDa for both nanorods coatings, while the median molecular weight values were 45 kDa for PSS and 48 kDa for PDADMAC. For PEGylated nanorods, the mean (72 kDa) and median (68 kDa) protein size was slightly higher. In addition, 40% of proteins identified in all four experiments bound to both negatively and positively coated nanoparticles (Table 1).

Based on the modest effects that the PEGylated nanorods had on fibroblast-mediated gel contraction and gene expression, the protein adsorption characteristics of these particles were also examined to determine if the minimal changes in gel contraction observed were due to the non-fouling and minimally adsorptive characteristics of the PEG coating. As shown in Figure 4, PEGylated nanorods do adsorb several proteins in the molecular weight ranges that were observed for both the PSS and PDADMAC coated particles. This observation is in accord with previous work that shows substantially reduced (but still detectable) protein adsorption to colloidal PEGylated particles compared to uncoated particles [36]–[39]. Identification of the proteins adsorbed onto the PEGylated nanorods revealed that despite the observed banding pattern, these particles adsorbed fewer proteins compared to the PSS and PDADMAC coated nanorods (Figure 5, Table S1). Fifteen unique proteins were identified and of these, only six were found in multiple screens (Table 2). In addition, most of these six proteins were also identified as having bound to the PSS and PDADMAC coated particles. Only one protein of rat origin, albumin, was identified.

**Table 2:** Identification of Proteins Adsorbed onto PEGylated Nanorods.

PEGylated coated Nanorods
α-1 acid glycoprotein
α-1 antitrypsin
α-2-HS glycoprotein
Albumin
Hemoglobin alpha chain
Serotransferrin/Transferrin

Proteins are listed in alphabetical order and are only listed if they were detected in all experiments (n = 4). Proteins shown in italics were identified as rat proteins (from the neonatal rat cardiac fibroblast cells) while all other proteins listed were of bovine origin.

### The Biological Connection between Isolated Proteins and Cell-Level Effects

An examination by species of origin indicated that most of the proteins identified as being associated with the nanorods were of bovine origin, and therefore due to the serum used in the culture media. However, biglycan was of rat origin, therefore produced by the fibroblasts, and was found in the protein fractions binding to both cationic and anionic nanorods but not PEGylated particles. Biglycan is a small ECM proteoglycan found in most connective tissues, and it is known to influence cell-ECM interactions and to modulate cell bioactivity by binding collagen and multiple growth factors, including members of the transforming growth factor β (TGFβ) superfamily [40], [41]. The core protein of biglycan is zwitterionic at neutral pH and could associate with either a cationic or anionic particle; but biglycan also contains 2 carbohydrate side chains composed of sulfated glycosaminoglycans. The high negative charge density of these side chains is favorable for coordinating with the positively charged surface of PDADMAC-terminated nanorods. As the PEGylated nanorods did not bind biglycan, this would suggest that sequestration of this proteoglycan by nanorods plays a key role in the observed biological effects. Recently, biglycan has been found in the sera of cancer patients (but not healthy volunteers) and is up-regulated in tumor endothelial cells compared to normal endothelial cells in vitro, and is produced in vivo in tumor blood vessels but not in normal blood vessels [42].

The local concentration of biglycan in the heart has been shown to influence cardiac fibroblast development in 6-week-old mice (in contrast to the neonatal rat cardiac fibroblasts used here) [43]. Biglycan deficiency at the organismal level of the mouse leads to a phenotypic switch of isolated cardiac fibroblasts toward myofibroblasts, which includes an increase in the cells’ ability to contract collagen gels, and an increase of α-SMA expression compared to wild-type mice. In our previous experiments, the presence of the nanorods decreased the cells’ ability to contract collagen gels and decreased α-SMA expression [28]. If the nanorods solely functioned as “molecular knock-outs” for biglycan, making it less bioavailable for the cells per the mice studies, then our results are directly opposed to the reported in vivo studies. However, comparing organisms of different species and of different ages, coupled with the heterogeneity of fibroblasts across tissues and even within the same tissue, makes direct comparisons of up-regulation and down-regulation less straightforward [44]. One possibility is that the cells in the presence of nanomaterials up-regulated biglycan itself, leading to its overproduction and hence isolation on the surface of the nanorods, along with the concomitant decreased cellular contraction ability and decreased α-SMA expression. Another possibility is that adsorbed biglycan does not function in the same way as free biglycan, its bioactivity altered or masked by association with the nanorods. Additionally, it is possible that the nanoparticles themselves, due to their high charge density, function as glycosaminoglycans mimetics, sequestering other proteins, such as fibroblast growth factor, thereby regulating fibroblast transformation into myofibroblasts [53], [54]. Experiments to test these hypotheses are currently in progress.

### Comparison of Protein Adsorption Data to Other Works

A number of investigators have begun to examine the role that protein adsorption onto nanomaterials has on biological effects of nanomaterials [24], [27], [28]. Lundqvist et al. studied the hard protein corona formed on both positively charged (amine modified) and negatively charged (carboxyl modified) polystyrene nanoparticles in human plasma [18]. They observed the binding of the same groups of serum proteins including Ig fractions, apolipoproteins, proteins involved in the complement pathway, and acute phase proteins. They found that only a small fraction of the proteins identified (5–10%) were common between the charged particles for both the 50 nm and 100 nm polystyrene nanoparticles. We have a similar result in that only a fraction of proteins found on the nanorods are common across the two charged particles. A recent meta-analysis of the nanoparticle “adsorbome” from 26 independent studies shows 125 adsorbed plasma proteins found across many types of nanoparticles [39]. Many of these proteins (serum albumin, transferrin, α-1-acid glycoprotein I) were found by us as well. However, once adsorbed onto the nanoparticles, the protein’s shape, structure and function may be altered due to possible conformational changes of the protein on the nanoparticle curved surfaces [31], [45]–[51]. Therefore, not only are the nanoparticles adsorbing proteins the cells might need for ECM remodeling; the nanoparticles could also be changing the activity of the proteins, rendering them “hyperactive” or inactive, even if the cells could access them while adsorbed.

## Conclusions

The results of this study offer insight into a possible mechanism by which polyelectrolyte-coated nanorods disrupt the matrix remodeling behavior of cardiac fibroblasts. The ability to bind proteins on the surface of the nanomaterials which can modulate the ECM remodeling behavior of fibroblasts suggests that nanoparticles could serve as novel therapeutic agents in the regulation of wound healing. If the protein adsorption process could be tailored for particular proteins, one could envision regulation of cell behavior by colloidal nanomaterials, by analogy to how fixed nanostructured substrates guide cell behavior.

## Supporting Information

Figure S1 **Size Distribution of Adsorbed Proteins.** PEG-, PSS-, and PDADMAC- coated nanorods showed a similar distribution of bound proteins based upon the protein size.
(JPG)

Table S1 **Complete list of unique proteins identified which bound to PSS-, PDADMAC-, and PEG-coated nanorods.** Three proteins identified as binding to PDADMAC-coated nanorods were of human origin and discounted as contamination since no human products were added to the cell culture media.
(DOCX)
