# Supplementary figures and images for: Adsorption of Cellular Proteins to Polyelectrolyte-Functionalized Gold Nanorods: A Mechanism for Nanoparticle Regulation of Cell Phenotype?
Source: PLoS One. 2014 Feb 6;9(2):e86670. doi: 10.1371/journal.pone.0086670 (PMC3916299; doi:10.1371/journal.pone.0086670)

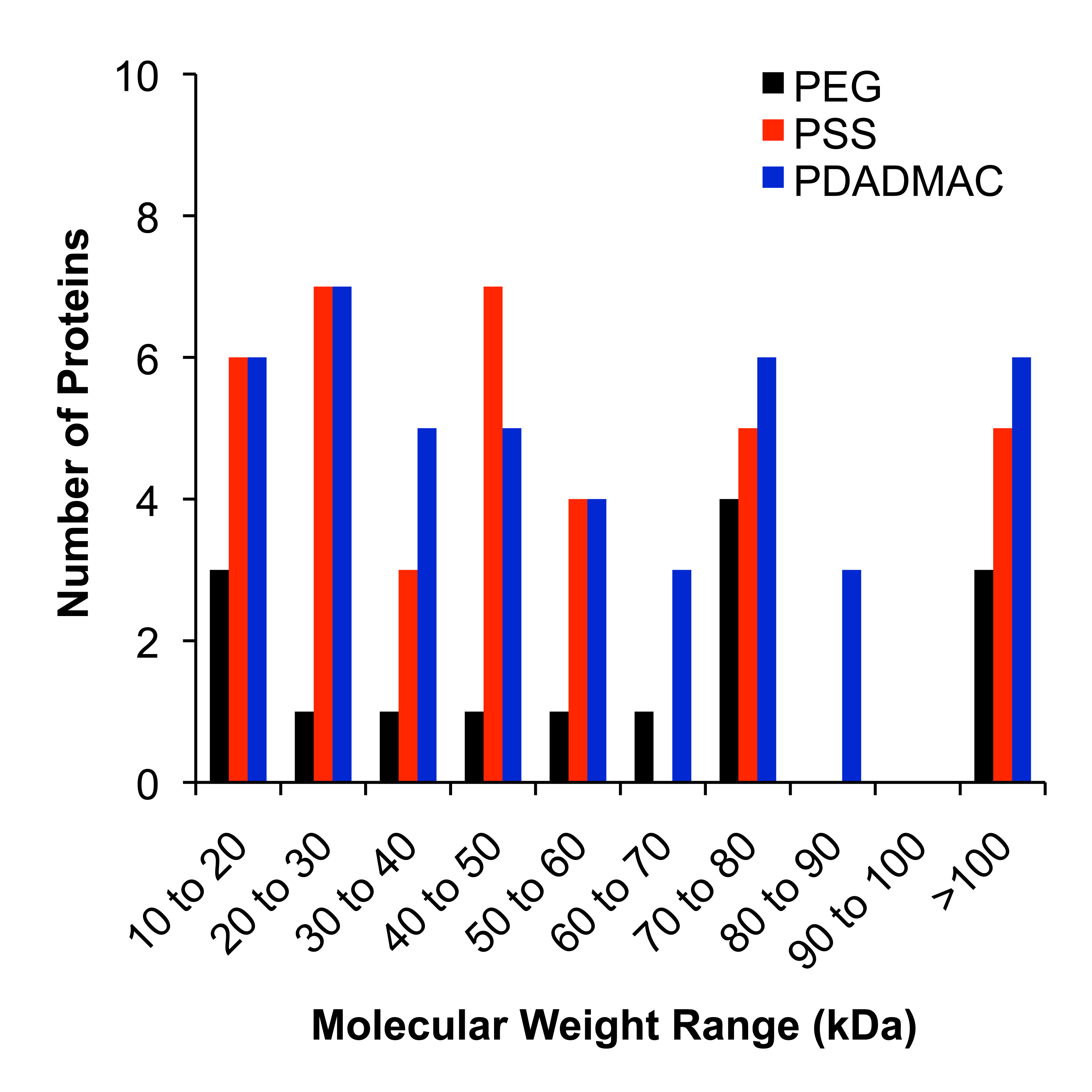

Supplement: Figure S1 — Size Distribution of Adsorbed Proteins. PEG-, PSS-, and PDADMAC- coated nanorods showed a similar distribution of bound proteins based upon the protein size. (JPG) [file pone.0086670.s001.jpg]
