# Supplementary material for: Adsorption of Cellular Proteins to Polyelectrolyte-Functionalized Gold Nanorods: A Mechanism for Nanoparticle Regulation of Cell Phenotype?
Source: PLoS One. 2014 Feb 6;9(2):e86670. doi: 10.1371/journal.pone.0086670 (PMC3916299; doi:10.1371/journal.pone.0086670)
Supplement: Table S1 — Complete list of unique proteins identified which bound to PSS-, PDADMAC-, and PEG-coated nanorods. Three proteins identified as binding to PDADMAC-coated nanorods were of human origin and discounted as contamination since no human products were added to the cell culture media. (DOCX) [file pone.0086670.s002.docx]

| **PEG** | **PSS** | **PDADMAC** |
| --- | --- | --- |
| Bovine serum albumin | α-2-HS-glycoprotein | Bovine serum albumin |
| α-2-HS glycoprotein | Bovine serum albumin | α-2-HS-glycoprotein |
| α-1 acid glycoprotein | Serotransferrin/Transferrin | Serotransferrin/Transferrin |
| α-1 antitrypsin | Complement factor H | Anti-testosterone antibody |
| Serotransferrin/Transferrin | Thrombospondin 1 | Calmodulin-like protein 5 |
| Hemoglobin alpha chain | Anti-testosterone heavy chain | Biglycan |
| α-2-macroglobulin | α-1 acid glycoprotein | Ig heavy chain precursor |
| Complement factor H | β-2-glycoprotein 1 | α-1 acid glycoprotein |
| α-fetoprotein | IgM heavy chain constant region | Vitamin D-binding protein |
| Prealbumin/transthyretin | α-2 macroglobulin | Immunoglobulin heavy chain constant region |
| Prepro complement component C3 | Vitronectin | α-2 macroglobulin |
| Hemoglobin fetal subunit beta | Biglycan | apolipoprotein A-II |
| Albumin | Complement C1q subcomponent subunit B | Dsp protein |
| Complement component C4 | Complement C1q subcomponent subunit A | Lactoferrin |
| Vitamin D-binding protein | α-1-B glycoprotein | α-1 antitrypsin |
|  | C-reactive protein | Hemopexin |
|  | Vitamin D-binding protein | Apolipoprotein H |
|  | Preprothrombin/prothrombin | Glyceraldehyde-3-phosphate dehydrogenase |
|  | IgG3 hey chain constant region | Zn- α2-glycoprotein |
|  | Complement C1q subcomponent subunit C | Junction plakoglobin |
|  | Prepro complement component C3 | Complement factor B |
|  | α-1 antitrypsin | Immunoglobulin heavy chain variable region |
|  | Similar to pro alpha 1(I) collagen | Thrombospondin 1 |
|  | Platelet factor 4 protein | Albumin |
|  | Serpin peptidase inhibitor, clade A, member 3 | IgG1 heavy chain constant region |
|  | Complement component 4 binding protein, alpha | Immunoglobulin light chain, lambda gene cluster |
|  | Complement component 1, r subcomponent | Complement C1q subcomponent subunit A |
|  | Immunoglobulin light chain, Lambda gene cluster | IGK protein |
|  | Immunoglobulin heavy chain constant region | Calcium homeostasis endoplasmic reticulum protein |
|  | Apolipoprotein A-II | α-fetoprotein |
|  | Complement component 3 | Serpin peptidase inhibitor, clade A, member 3 |
|  | Fetuin B | Hemoglobin fetal subunit beta |
|  | Prealbumin/transthyretin | Hemoglobin alpha chain |
|  | Hemoglobin alpha chain | AMBP protein |
|  | Hemoglobin fetal subunit beta | Adiponectin |
|  | α-fetoprotein | Thrombospondin-4 |
|  | Leptin | Fibulin-1 |
|  |  | LOC498793 protein |
|  |  | Galectin-3-binding protein precursor |
|  |  | Vitronectin |
|  |  | Hemoglobin subunit alpha |
|  |  | Inter-alpha-trypsin inhibitor heavy chain H2 |
|  |  | Apolipoprotein A-I |
|  |  | Prealbumin/transthyretin |
|  |  | Serum albumin precursor |
|  |  | Prepro complement component C3 |
|  |  | C-reactive protein precursor |
|  |  | Complement component C4 |
